# Molecular phylogeny supports S-chaetae as a key character better than jumping organs and body scales in classification of Entomobryoidea (Collembola)

**DOI:** 10.1038/srep12471

**Published:** 2015-07-27

**Authors:** Feng Zhang, Dan-Dan Sun, Dao-Yuan Yu, Bei-Xin Wang

**Affiliations:** 1 Department of Entomology and Key Laboratory of Integrated Management of Crop Diseases and Pests, College of Plant Protection, Nanjing Agricultural University, Nanjing 210095, P. R. China; 2 Department of Ecology, College of Resources and Environmental Sciences, Nanjing Agricultural University, Nanjing 210095, P. R. China

## Abstract

The jumping organ (furcula) is the most characteristic structure among collembolans, and it is of great taxonomical values at higher levels. The largest superfamily Entomobryoidea is traditionally classified into four families only by the morphology of the furcula. Actually, many taxa among these families are strikingly similar in morphology without considering furcula. The phylogeny of Entomobryoidea was reconstructed here based on mitochondrial and ribosomal fragments. This indicated that both Paronellidae and Cyphoderidae were ingroups within Entomobryidae with the former polyphyletic. Topology tests, which used the likelihood and Bayesian approaches, also rejected the traditional hypotheses relying on furcula morphology. Further ancestral state reconstructions have revealed that traditional taxonomical characters, i.e., furcula and body scales, had multiple independent origins in Entomobryoidea whereas tergal specialized chaetae (S-chaetae) exhibited strong phylogenetic signals. By integrating both molecular and morphological evidence, the results of this study drastically undermine the present classification of Entomobryoidea. Tergal S-chaetotaxic pattern in combination with other characters are more reasonable in taxonomy at suprageneric levels than convergent furcula. This study provides new insights of the jumping organ, which could be adaptively modified during evolution of Collembola.

The jumping organ or furcula is perhaps the most characteristic feature of Collembola (Fig. 1). Furcula has two main functions: to make jump and to escape from predators^1^. The furcula originates from a pair of appendages on the fourth abdominal segment (Abd.), with the basal part fused to form the manubrium and the two distal parts separated and developed into dens (Fig. 1a), whose most distal parts bear a small mucro^2^,^3^ (Fig. 1b–f). All three parts of furcula are of great taxonomical values from specific to familial levels in the traditional classification. In addition, distribution and morphology of body scales are also important diagnostic characters at generic and suprageneric levels. However, they rarely have been studied within Collembola in an evolutionary view.

More than one fourth of collembolan species belong to superfamily Entomobryoidea possessing a well-developed furcula^4^. Börner divided Entomobryidae sensu Entomobryoidea Szeptycki, 1979^5^ into Entomobryinae, Cyphoderinae, and Paronellinae, with the last having uncrenulate dens^6^ (Table 1). Later, Absolon and Ksenemann raised three subfamilies to families, separating Paronellidae into scaled Paronellinae and unscaled Salinae and included oncopodurines in Cyphoderidae^7^. Yosii analyzed the phylogenetic significance of chaetotaxy in Collembola and treated Cyphoderinae sensu Börner as a family^8^. Both Szeptycki^5^ and Deharveng^9^ treated Börner’s subfamilies as families, together with the fourth small family Microfalculidae having no mucro. The only difference from the classification of Absolon and Ksenemann was that Oncopoduridae was excluded from Entomobryoidea. In 2008, Soto-Adames *et al.* demoted Cyphoderidae to a subfamily within Paronellidae because of differences from the other subfamily Paronellinae in the presence of fringed dental scales^10^ (Fig. 1h). Actually, most researchers have preferred treating cyphoderids as a separate family due to their elongate mucro as well as the absence of eyes and pigment. Geographical distribution may also provide potential discrimination evidence, the cyphoderids and entomobryids worldwide, but most genera and species of Paronellidae *s. s*. distributed in tropical and subtropical zones^11^,^12^.

Szeptycki proposed the modern classification of Entomobryoidea based on his great contribution on the evolution of tergal chaetotaxy; whereas he pointed out that phylogenetic relationships between taxa within Entomobryoidea remained obscure^5^. Paronellidae and Cyphoderidae distinctly related to higher Entomobryidae (Entomobryinae, Seirinae, and Lepidocyrtinae) due to the elongation of the fourth abdominal segment. These three groups are very similar and cannot be distinguished even after rigorous examination of synapomorphies. The only separating criterion is dens (middle part of furcula), which is smooth and nearly cylindrical in Paronellidae while crenulate and strongly taped (Fig. 1a) in Entomobryidae^10^. However, confusion is brought by the Paronellidae genera *Akabosia*^13^ and *Yosiia*^14^, which have distinct crenulations on their dens (Fig. 1c) as those in Entomobryidae. A new genus (unpublished data), which formally belonged to Paronellidae and has crenulations on the distal part of dens and reduced mucro, challenges the traditional view too. Yoshii introduced the remarkable coincidence between Entomobryidae and Paronellidae taxa based on scale morphology and macrochaetotaxy^15^. All of the above evidence strongly questions the monophyly of Paronellidae and the present classification of Entomobryoidea.

Molecular phylogeny of Entomobryoidea has been rarely studied in the past, usually sampling few entomobryid taxa for the analyses^16^,^17^,^18^,^19^. Xiong *et al.* sampled a paronellid species (*Callyntrura* sp.) in the phylogeny of Collembola based on rRNA genes^17^; however, this taxon was finally located within Entomobryidae and sistered to three sampled Entomobryinae species upon the trees, but never departed from Entomobryidae. Other studies mainly focused the largest family Entomobryidae within the superfamily. Zhang *et al.*^20^ reconstructed the phylogeny of Entomobryidae based on the nuclear 18/28S rRNA and the mitochondrial 16S rRNA, indicating the independent origins of body scales. Subsequently, Zhang and Deharveng^21^ discovered the great phylogenetic values of tergal specialized chaetae (S-chaetae) in Entomobryidae and further revised the family. S-chaetae are smooth, blunt, more translucent under light microscope, and differ from ordinary chaetae (Fig. 1a,g). The absence of plurichaetosis, the intraspecifically stability with development, and the variety of the pattern between taxa make S-chaetotaxy a promising character for taxonomy^9^. S-chaetotaxy has been widely used in the taxonomy of Isotomidae^22^, but it was rarely explored in the phylogeny of higher levels except Poduromorpha and Entomobryomorpha^23^.

To improve the understanding of the jumping organ and clarify the evolutionary relationships among Entomobryoidea, this study reconstructed the phylogeny based on the mitochondrial and nuclear genes by likelihood and Bayesian algorithms. Several monophyletic hypotheses were assessed by using both likelihood and Bayesian approaches. Furthermore, ancestral character states and phylogenetic signals of the jumping organ and other potential useful characters, i.e., body scales and S-chaetae, were also examined upon phylogenies reconstructed here.

## Results

### Phylogenetic inference

Maximum likelihood (ML) and Bayesian inference (BI) analysis generated the same results at the suprageneric levels. Phylogeny of five main clades was reconstructed well with high Bayesian posterior probabilities (BPP) (>0.98, Fig. 2): (Orchesellinae + (Heteromurinae + (“Lepidocyrtinae” + (Seirinae + “Entomobryinae”)))). Monophyly of Heteromurinae and the sister relationship of Seirinae and “Entomobryinae” possessed slightly weak bootstrap support (maximum likelihood bootstrap (MLB) >0.65). Some “Entomobryinae” clades of very low MLB values in ML-analysis appeared in a polytomy in BI consensus tree.

Monophyly of Paronellidae *s. s.* and Paronellidae *s. l*. (Paronellidae + Cyphoderidae) taxa was never recovered, while Orchesellinae and Heteromurinae taxa were always located at the root of the ingroup. In all analyses, seven Paronellidae species were located within Entomobryinae (labeled as “Entomobryinae” in Fig. 2). One Paronellidae and one Cyphoderidae within Lepidocyrtinae (labeled as “Lepidocyrtinae” in Fig. 2). *Akabosia matsudoensis*, which bears crenulate dens and was placed in Paronellidae, was sistered to two other Cremastocephalini taxa. Four Callyntrurini taxa within “Entomobryinae” never formed a monophyletic clade.

### Tree topology comparison

Both approaches absolutely rejected the hypotheses B and C (Table 2), which indicated that Paronellidae and Paronellidae sensu Soto-Adames *et al.* were polyphyletic as ingroup of Entomobryidae. Hypotheses D (monophyly of Orchesellinae *s. l.*), E (Heteromurinae as the basal group) and F (Seirinae sistered to “Lepidocyrtinae”) were accepted by CONSEL test (p > 0.05) but were rejected by Bayes factor test (3 < BFs < 5).

### Phylogenetic signal

The retention index (ri) and method of Maddison and Slatkin^24^ were employed for the tests of phylogenetic signal. No significant difference was observed between analyses on ML and BI trees (Table 3). The furcula (dens plus mucro) and body scales indicated the weak phylogenetic signals with low ri values (<0.65), and the relative large ratio of observed/permutation number of character transitions (obs/permu > 0.65), while tergal S-chaetae exhibited as a non-homoplastic character (ri = 1 for ML tree). When S-chaetae on each tergum were separately analyzed, all indicated strong phylogenetic signals (ri > 0.85, obs/permu < 0.45).

### Ancestral state reconstruction

Ten well supported deep nodes (BPP > 0.98, Fig. 3) were selected for ACSR of eight observed characters. Posterior probabilities (PP) of ancestral states under maximum parsimony (MP), ML, and Bayesian (BayesTraits) methods were summarized in Supplementary Table S2. The results of MP and ML generally agreed very well in most nodes. The results of Bayesian single-rate and unrestricted-rate models sometimes provided strikingly different probabilities of ancestral states for body scales and S-chaetae on Abd. I (Supplementary Table S2). The single-rate Bayesian model performed closer to ACSR of MP and ML. The logarithm difference in harmonic mean likelihood between two rate models was always less than two, indicating no significantly differences between two models.

For the jumping organ, ancestral state of nodes 6–10 was dens crenulate and mucro bidentate (most PP > 0.95, Fig. 3, Supplementary Table S2). Taxa bearing smooth dens, or non-bidentate mucro, or body scales, never formed a monophyletic group. Smooth dens appeared independently in Entomobryoidea at least five times, non-bidentate mucro five times, body scales nine times.

For the tergal ordinary S-chaetae on ancestral states (node 10, all PP > 0.9), the S-chaetae were 2, 2 on thoracic segment (Th.) II and III as well as 1 on abdominal segment (Abd.) I (Supplementary Figs S1, S2). They were transformed into states 1, 1 on thorax and 0 on Abd. I three times, once in Lepidocyrtinae, once in Seirinae, and once in the clade of ((*Akabosia *+ *Salina*) + *Callyntrura*). The ancestral state of the S-chaetae on Abd. II, III, and V was likely to be multi-setaceous (>4) type (Bayesian PP > 0.5). When the tergal S-chaetae was analyzed as a single character, the ancestral states were equivocal at nodes 7–10 by using ML-ACSR (Fig. 4b), and dispersed much of the PP on several states under BI-ACSR (Supplementary Table S2).

## Discussion

**Figure 1:**
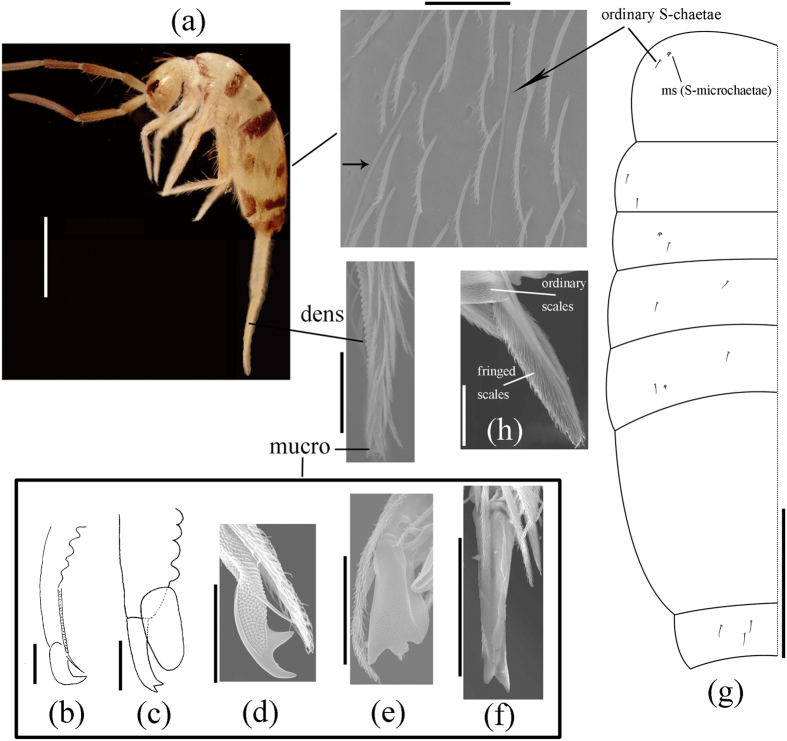
Jumping organ and tergal chaetae in Entomobryoidea. (**a**) *Homidia sinensis* Börner (Entomobryidae), crenulate dens and smooth specialized chaetae (S-chaetae) on the fourth abdominal segment; (**b**–**f**) types of mucro; (**b**) *Coecobrya caledonica*, falcate; (**c**) *Akabosia matsudoensis*, bidentate; (**d**) *Lepidocyrtus felipei*, bidentate; (**e**) *Salina pictura*, tridentate; (**f**) *Callyntrura guangdongensis*, two apical and three large and one minute lateral teeth; (**g**) schema of tergal S-chaetae in *Homidia sinensis*, chaetal formula 2, 2/1, 2, 2, ?, 3 (S-microchaetae excluded); and (**h**) fringed dental scales in *Cyphoderus javanus*. Scale bars: (**a**) 200 μm for S-chaetae and 500 μm for others; (**b**–**f**) 50 μm; (**g**) 300 μm; (**h**) 10 μm.

**Figure 2:**
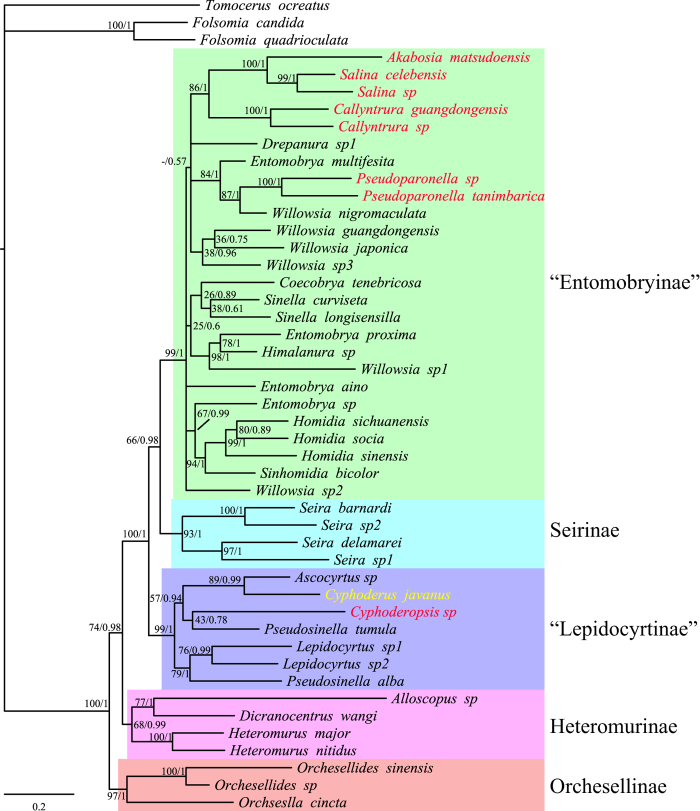
Bayesian phylogeny of Entomobryoidea based on molecular dataset. Node values represent likelihood bootstrap and posterior probabilities, respectively, with a – indicating nodes not compatible between the analyses. Paronellidae terminals are marked as reddish ones, Cyphoderidae as yellow one, and others as Entomobryidae. “Entomobryinae” and “Lepidocyrtinae” indicate the group contains paronellid and cyphoderid taxa besides traditional taxa.

Primary classification framework of Entomobryoidea has not been changed since Börner^6^, which separated the superfamily into three main groups (Table 1). The furcular dens are crenulate (Fig. 1a) in Entomobryidae, smooth in Paronellidae, and smooth with fringed scales (Fig. 1h) in Cyphoderidae. Molecular phylogeny reconstruction and tree topology tests did not support the monophyly of Paronellidae *s. s*. and Paronellidae *s. l*. (Paronellidae + Cyphoderidae), which were treated here as the ingroup of Entomobryoidea (Fig. 2). The results of this study fairly demonstrated Szeptycki’s doubt^5^ by molecular approaches, and drastically undermined the traditional classification of the superfamily.

The separation of crenulate and smooth dens is usually available in the morphology for Entomobryoidea except *Yossia* and *Akabosia*, the latter genus possessing crenulate dens but distally large bladder-like appendage and elongate mucro with apically bidentate (Fig. 1c). Molecular phylogeny clustered *Akabosia* and *Salina* together with absolute high node support (100/1, Fig. 2), which again demonstrated the viewpoint of Kang and Park^25^ based on morphology. Tergal S-chaetotaxy (Fig. 4b), discrete eyes in appearance, and large tenent hairs also support a closer relationship between them besides several other distinguishable characters mentioned by Kang and Park. The systematic position of *Akabosia* indicates that Paronellidae taxa could bear both crenulate and smooth dens.

**Figure 3:**
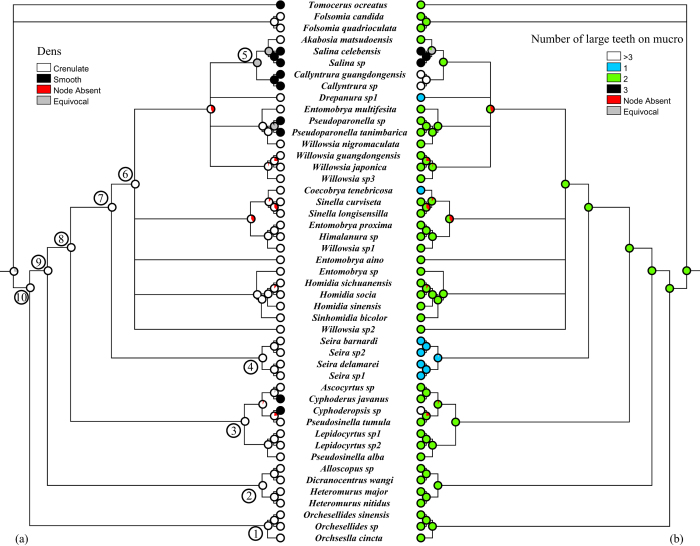
Evolution of jumping organ in Entomobryoidea. (**a**) dens; and (**b**) mucro. ACSR was reconstructed over 15,000 posterior trees using ML method and shown on a Bayesian consensus tree. Each node indicates character states with different colorations and the proportion of the state over all examined trees. Character states and their coding numbers are shown. Ten well-supported deep nodes for which ACSR were performed are also indicated.

**Figure 4:**
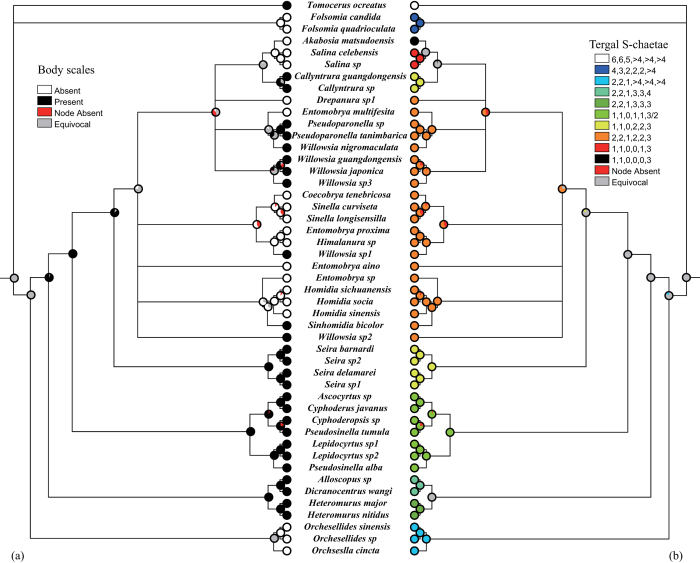
Evolution of body scales and tergal S-chaetae in Entomobryoidea. (**a**) body scales; and (**b**) tergal S-chaetotaxic pattern. ACSR was reconstructed over 15,000 posterior trees using ML method and shown on a Bayesian consensus tree. Each node indicates character states with different colorations and the proportion of the state over all examined trees. Character states and their coding numbers are shown.

The mucro is usually variable (Fig. 1b–f) at the generic levels in Entomobryomorpha, such as those in Isotomidae^22^. “Highly variable mucro” is almost impossible to be accurately defined as a synapomorphy for Paronellidae taxa (Fig. 3b). Body scales are also of different origins for the *Callyntrura* and *Pseudoparonella* (Fig. 4a), as well as those in Entomobryinae^20^.

Actually, the corresponding groups of Paronellidae, Cyphoderidae, and Entomobryidae have great morphological similarities without considering the furcula^5^. Yoshii’s findings^15^ of the coincidence between Paronellidae and Entomobryidae partially supported the present molecular phylogeny; one character (tergal macrochaetae in his table) is homoplastic in the traditional view, but it is consistent here. Besides the elongated fourth abdominal segment (Fig. 1), Paronellini, Bromacanthini, and Cyphoderidae have the presence of body scales with fine ciliations as well as reduced cephalic and tergal macrochaetae with developed bothriotrichal complexes^11^,^15^,^26^,^27^, which are also the representative features for Lepidocyrtinae^5^. Tergal S-chaetotaxy 1, 1/0, 1, 1 from mesothorax to Abd. III is also a potential synapomorphy for “Lepidocyrtinae” (Fig. 4b). Dental morphology is unavailable for the separation of *Salina*/*Akabosia*/*Callyntrura*/*Pseudoparonella* and Entomobryinae, so that no reliable characters could be used for their classification.

As discussed above, traditional characters, such as furcula, body scales, etc., are no longer suitable for the classification of Entomobryoidea at the familial level. Low phylogenetic signals (ri < 0.65, Table 3) also implied their high homoplasy. Alternatively, tergal S-chaetae, whatever combined or separate analyses, exhibited a much stronger phylogenetic signal (ri > 0.85, Table 3), performing perfectly at deep levels (Fig. 4b). Multiple patterns in monophyletic “Entomobryinae” were mainly resulted from those Paronellidae taxa, four sampled genera bearing four patterns. As for unsampled Microfalculidae with mucro absent and dens crenulate, the morphological examination revealed that reduced S-chaetotaxy and tergal macrochaetae similar to *Akabosia*, strongly developed tenent hairs, and discrete eyes. Both groups live in the very humid epigeic environment, such as on leaves or barks, further implying that Microfalculidae might be the derivative of *Akabosia*/*Salina* (personal communication with C. D’Haese).

Early taxonomical context in Cyphoderidae was comprised of Cyphoderini and Troglopedetini (the latter is now synonymized with Paronellini^26^) for eyes reduced and body scales present^6^,^28^. Later, Troglopedetini was transferred to Paronellidae due to the absence of fringed dental scales^5^,^7^,^9^,^10^. However, molecular phylogeny (Fig. 2) supports the closer relationship of *Cyphoderus* (Cyphoderini) and *Cyphoderopsis* (Troglopedetini), both of which have been clustered with Lepidocyrtinae. Szeptycki^5^ noticed that the great similarity in chaetotaxy between *Cyphoderus* and Lepidocyrtinae. In addition, a second pair of bothriotricha on the antero-lateral head was described in *Cyphoderopsis*^29^ and Cyphoderidae^30^ (*Cyphoderus*, *Troglobius*), also implying their resemblance. The cyphoderids may have derived from a *Cyphoderopsis*-like ancestor for the elongate mucro. Because of its blindness and fringed dental scales, Cyphoderidae is possibly monophyletic but inappropriate to be treated as a separate family. Comprehensive comparative studies of “Lepidocyrtinae”, including Lepidocyrtinae, Paronellini (Troglopedetini), Bromacanthini, and Cyphoderidae, may help to improve the understanding of the final position of cyphoderids.

Orchesellinae *s. l.* (Orchesellinae + Heteromurinae) was considered to be the most primary subfamily within Entomobryidae for the non-elongate Abd. IV^5^. Zhang *et al.*^20^, and this study achieved the consistent results, supporting the separation of sampled taxa into unscaled and scaled groups. The S-chaetotaxic pattern also confirmed this separation^21^ (Fig. 4b). However, previous studies have not resolved the relationship between Orchesellinae and Heteromurinae. The present analyses placed Orchesellinae in a more basal position (Fig. 2). Test by Bayes factors gave the strong evidence against the two hypotheses, whereas CONSEL likelihood test could accept the alternative hypotheses (Table 2). Sister relationships of Heteromurinae and non-Orchesellinae taxa were positively validated by relatively high node support (74/0.98) and by Bayes factor tests.

When looking at the scaled Orchesellinae, *Alloscopus*, a subgenus of *Heteromurus* with five antennal segments, was sistered to *Dicranocentrus* of six antennal segments with high support (Fig. 2). In the morphology, the four S-chaetae on Abd. V also supported the closer relationship of *Alloscopus* and *Dicranocentrus* (Fig. 4b). The present phylogeny again rejects the traditional classification of applying the number of antennal segments. Moreover, *Alloscopus* (mainly Southeast Asia^31^) and *Dicranocentrus* have a tropical distribution, and *Heteromurus* are mostly in the Holarctic area^32^.

Previous studies supported a closer relationship between Seirinae and Lepidocyrtinae^5^,^8^,^10^. Zhang *et al.*^20^ grouped Seirinae and Lepidocyrtinae but with weak support (45/0.67), while CONSEL topology tests rejected the alternative hypothesis of a sister relationship between the Seirinae and Entomobryinae. However, the phylogeny reconstructed in this study indicated Seirinae is closer to “Entomobryinae” than “Lepidocyrtinae” (Fig. 2). Compared to the reconstructions of Zhang *et al.*^20^, this study sampled more taxa (Paronellidae, Cyphoderidae) and sequenced one more mitochondrial marker COI, resulting in higher resolution and support at deep nodes. Zhang *et al.*^20^ mentioned some features similar to Entomobryinae, such as polymacrochaetotaxic chaetotaxy. Another notable coincidence, *Seira* (Seirinae) and *Callyntrura* (“Entomobryinae”) bear the same S-chaetotaxic pattern 1, 1/0, 2, 2, ?, 3 in addition to their distribution (tropical and subtropical area^11^,^33^). Actually, CONSEL topology tests accepted the possibility of the traditional hypothesis, although the Bayes factor gave contrary evidence (3 < ΔBFs < 5, Table 2). By considering the great similarity in morphology, the hypothesis cannot be rejected that the Seirinae and Lepidocyrtinae are sister groups.

As the oldest hexapods^34^, Collembola possesses the characteristic jumping organ. The basic structure (manubrium, dens, and mucro) is highly diversified at all levels, even completely absent in some Poduromorpha and Isotomoidea^4^. Because the smooth (that is not crenulate) dens usually occur in Poduromorpha and in many more primitive Isotomoidea, so then the absence of crenulations was considered to be primitive (plesiomorphic) for Collembola^35^. However, this viewpoint was not validated in Entomobryoidea, which have crenulate dens occurring at all deep nodes (6–10) in ACSR (Fig. 3a, Supplementary Table S2). It is not a surprising result because Entomobryoidea may originate from higher Isotomoidea-like ancestors having long and crenulate dens^5^. Smooth dens are an apomorphic trait during evolution of Entomobryoidea, and they independently appeared at least four times. The exact reason is unknown why the crenulate dens are transformed into the smooth one. Evolution of furcula is very likely to be related to the mechanism of jumping. Among Entomobryoidea, smooth dens are possibly more adapted to the wet and warm microenvironments, so that most Paronellidae taxa who live in the tropical and subtropical zones, often on the leaves^11^. Cyphoderidae, blind and edaphic, have similar dens to those edaphic primitive groups (most Poduromorpha and Isotomoidea). When thinking about *Akabosia*, its secondary crenulate dens may have transformed from ancestral smooth dens (node 5, high possibility supported by Bayesian analyses, Supplementary Table S2), which may be very likely due to its present temperate distribution (Japan, Korea, and northern China) rather than subtropical and tropical zone. Another furcular component mucro, variable among genera, has two common large teeth as many Isotomoidea in an ancestral state (Fig. 3b).

Taxonomical and evolutionary implications of tergal S-chaetae in Entomobryoidea were overlooked in previous studies. This study confirmed their strong phylogenetic signals among Entomobryoidea (Table 3). The evolution of tergal S-chaetae (S-microchaetae excluded) has a reduced tendency from low to high groups (Fig. 4b). Compared to Entomobryoidea, primitive Isotomidae (state 1) and Tomoceridae (state 0), which possess much more abundant S-chaetae, particularly on the Th. II-Abd. I segments with the greatest numbers. Among the Entomobryoidea, Orchesellinae, and Heteromurinae at the basal position have more S-chaetae (≥3) on Abd. II, III and V, as well as relatively higher numbers (2, 2, 1, Fig. 1g) on Th. II-Abd. I than those in other groups. Furthermore, their numbers among partial segments possibly correlate. For example, Th. II-Th. III-Abd. I have 2, 2, 1 in Orchesellinae *s. l*. and Entomobryinae, and one postero-lateral S-chaeta (acc. p6 in Szeptycki^36^) is absent for three segments in other groups. For Abd. II and III, the middle S-chaeta in Heteromurinae (3, 3) is missing in Seirinae and Entomobryinae (2, 2); both middle and lateral S-chaetae are lost in the “Lepidocyrtinae” (1, 1). This correlation occurs in the neighboring segments, which are supposed to be homologous in chaetotaxy and function and may involve with the origin of segments^5^. When ACSR of tergal S-chaetae was analyzed as a single character, ancestral states are equivocal at deep nodes 7–10, with state 2 (2, 2, 1, >4, >4, >4) preferred at node 10 (Fig. 4b, Supplementary Table S2). Separate analyses for each segment also provide the opportunities to trace their possible evolution mode (Supplementary Figs S1-5) and confirms the previous single analysis although the evolution of S-chaetae among different segments seems to be not independent. The ancestors of Entomobryoidea seem to have relatively high number of tergal S-chaetae like those in the Orchesellinae.

## Methods

### Taxa sampling

To avoid debates here, the Szeptycki’s classification^5^ on Entomobryoidea was adopted here, which recognized four families. One Tomoceridae and two Isotomidae species were chosen as the outgroup. Forty-four ingroup species covering the main groups of Entomobryoidea were selected for this study, respectively with 35 Entomobryidae (three Orchesellinae, four Heteromurinae, 19 Entomobryinae, four Seirinae and five Lepidocyrtinae), eight Paronellidae (three Cremastocephalini including problematic taxa *Akabosia matsudoensis*, four Callyntrurini, one Paronellini), and one Cyphoderidae species. Monospecific Microfalculidae from Africa was not included in the present analysis. Taxa names, traditional taxonomical position prior to this study, collection locality, and GenBank accession numbers are provided in Supplementary Table S1. All specimens were collected by aspirator or Tullgren-Berlese funnels, stored in 99% ethanol at –20 ˚C, and morphologically identified by using Nikon SMZ1000, Nikon 80i microscopes and a Hitachi scanning electron microscope (SEM).

### DNA extraction and sequencing

DNA was extracted by using a DNeasy Blood and Tissue Kit (Qiagen, Hilden, Germany) and following the manufacturer’s standard protocols. PCR amplification of the four fragments, mitochondrial COI, 16SrRNA (16S), nuclear 18SrRNA (18S), and 28SrRNA D1–3 (28S), was carried out by following Zhang *et al.*^20^,^37^. All PCR products were checked on a 1.0% agarose gel, purified and sequenced by Majorbio (Shanghai, China) on an ABI 3730XL DNA Analyzer (Applied Biosystems). Sequences were read and assembled in Sequencher 4.5 (Gene Codes Corporation, Ann Arbor, Michigan, USA), and were deposited in GenBank (Supplementary Table S1). Sequences were blasted in GenBank and checked for possible errors. They then were preliminarily aligned by using MAFFT v7.149 by the Q-INS-I strategy^38^. Alignments were checked and corrected manually. Partial ambiguous sites of 16S were excluded from all the analyses. In the final 4015 bp concatenated alignment, COI, 16S, 18S, and 28S were 658 bp, 416 bp, 1605 bp, and 1336 bp, respectively; a total of 332 sites were variable and parsimony uninformative, and 989 sites were variable and parsimony informative.

### Phylogenetic analyses

The partitioned dataset was analyzed by ML and BI. All three coding positions of protein-coding gene COI were included in the analyses. Best-fitting substitution models were assessed for each locus (partition) under the AIC criterion in jModelTest 2.1.4^39^, the TVM+I+Γ, TPM2uf+I+Γ, GTR+I+Γ and GTR+I+Γ models selected for COI, 16S, 18S and 28S, respectively. The former two models cannot be implemented in subsequent software, then an alternative GTR+I+Γ model was used. ML trees were reconstructed in raxmlGUI1.3^40^,^41^ with the GTRGAMMAI model and 1000 bootstrap replicates. BI-analyses were conducted in an online version of MrBayes 3.2.2^42^,^43^ with four chains (three heated, one cold) ran and the GTR+I+Γ model. Model parameters were unlinked and the model allowed the overall rate to be different across partitions. To avoid the problem of branch-length overestimation, the compound Dirichlet priors “brlenspr = unconstrained: gammadir (1, 1, 1, 1)” for branches lengths were incorporated^44^. The number of generations for the total analysis was set at 50 million, with the chain sampled every 5,000 generations. The burn-in value was 25% and other parameters were set as default options. To confirm convergence, the average standard deviation of split frequencies and the potential scale reduction factor values were visualized in MrBayes, and evaluating effective sample size values were checked in Tracer 1.5^45^.

### Tree topology comparison

Five topology hypotheses on constraining monophyly were tested under likelihood and Bayesian theory frameworks: A, best trees without any constraints; B, Entomobryidae + Paronellidae sensu Soto-Adames *et al.*^10^; C, Entomobryidae + (Paronellidae + Cyphoderidae); D, (Orchesellinae + Heteromurinae) + remaining taxa; E, Heteromurinae + (Orchesellinae + remaining taxa); F, “Entomobryinae” + (Seirinae + “Lepidocyrtinae”) (paronellid and cyphoderid taxa not excluded from three clades). Probability values (p-value) of approximately unbiased (AU) tests, Shimodaira-Hasegawa (SH) and weighted Shimodaira-Hasegawa (WSH) tests were calculated in CONSEL V0.1j^46^ with the default settings. Per-site log likelihoods prior to CONSEL analyses were generated by raxmlGUI. Hypotheses having p-values significant at the level of greater than 0.05 were rejected.

Evaluation of Bayes factors (BFs) has been a standard approach to perform model selection in Bayesian phylogenetics^47^,^48^. Marginal likelihood estimator by stepping-stone sampling^49^,^50^ was calculated in MrBayes for five hypotheses. Informed topology was strictly constrained in the prior because standard way of BF tests of monophyly can be misleading^51^. Markov chain Monte Carlo (MCMC) processes are the same as previous analyses (ngen = 50000000 samplefreq = 5000). A logarithm difference (logBF_1_-logBF_0_) in the range of three to five was considered to give strong evidence against hypothesis zero, while the difference value above five gives very strong evidence^52^.

### Phylogenetic signal tests

Several characters, which were important for taxonomy in Entomobryoidea or of potential phylogenetic significance, were selected for assessing phylogenetic signal: furcula (dens and mucro), body scales, and tergal ordinary S-chaetae (S-microchaetae excluded). S-chaetotaxic patterns on each segment (mesothorax, metathorax, and abdominal segments I, II, III, and V) were also separately analyzed. The S-chaetae on the fourth abdominal segment were not considered here because no simple pattern can be clarified, and most of them were often lost during specimen preparation. Character states and coding were shown in Supplementary Table S2.

The phylogenetic signal of morphological characters was assessed on a ML tree and a BI consensus tree by employing the retention index (ri) and the method of Maddison and Slatkin^24^. High ri values (≥0.85) indicated low homoplasy and a demonstrable phylogenetic signal. In the latter approach, the observed number of character transitions and permutation of character values were calculated in Mesquite. Relatively small observed number of transitions implied that the character evolved slowly enough to retain phylogenetic information^24^. All analyses were performed in Mesquite 2.75^53^.

### Character evolution

Results of the ancestral character state reconstructions (ACSR) were often strikingly different depending on the method used^54^. The maximum parsimony, maximum likelihood, and Bayesian methods were performed for the ACSR of deep nodes with high support (BPP > 0.95). By considering the uncertainty in the tree topology and branch lengths, all analyses were reconstructed on 15000 Bayesian posterior trees and summarized on a BI consensus tree. The MP- and ML-ACSR were calculated in Mesquite. ML reconstructions were performed under a single-rate Mk1 likelihood model^55^. Fully Bayesian reconstructions were performed by using BayesTraits V2.0 (Beta)^56^,^57^, for the two strategies employed in this study. The first analysis used a reverse jump (rj) MCMC method on an unrestricted model to integrate over model parameters and model restrictions. The second analysis used rj-MCMC on a single-rate model by constraining all transformation rates to be equal. Both analyses employed a hyper prior approach to seed the mean and variance of the gamma prior from uniform hyper priors both on the interval of zero to 10. All analyses were run for 50 million MCMC generations with the first 20% as burn-in and sampled every 5,000 generations. Each analysis was duplicated in order to check for convergence.

## Additional Information

**How to cite this article**: Zhang, F. *et al.* Molecular phylogeny supports S-chaetae as a key character better than jumping organs and body scales in classification of Entomobryoidea (Collembola). *Sci. Rep.* **5**, 12471; doi: 10.1038/srep12471 (2015).

## Supplementary Material

Supplementary Information

Supplementary Table S2

## Figures and Tables

**Table 1:** Classification systems and corresponding diagnostic characters used in Entomobryoidea.

**Börner**^6^	**Absolon & Kseneman**^7^	**Yosii**^8^	**Szeptycki**^5^	**Soto-Adames** ***et al***.^10^	**Diagnostic characters for dens**
Entomobryinae	Entomobryidae	Entomobryidae	Entomobryidae	Entomobryidae	Crenulate
Paronellinae	Paronellidae		Paronellidae	Paronellidae	Smooth^a^
Cyphoderinae	Cyphoderidae*	Cyphoderidae	Cyphoderidae		Smooth^b^
-	–	–	Microfalculidae	Microfalculidae	Mucro absent

a, smooth without fringed scales; b, smooth with fringed scales (Fig. 1h); *, oncopodurines included.

**Table 2:** Comparison of tree topology hypotheses by using likelihood and Bayesian approaches.

Hypotheses	**Likelihood Tests**			**Bayes Factors**	
	**AU**	**SH**	**WSH**	**Model likelihood**	**Logarithm difference**
**A**	0.789	0.961	0.968	−37383.64	0
**B**	4e-028**	0**	0**	−37797.09	413.45**
**C**	4e-034**	0**	0**	−37795.22	411.58**
**D**	0.227	0.681	0.49	−37387.47	3.84*
**E**	0.237	0.651	0.45	−37387.78	4.14*
**F**	0.374	0.714	0.631	−37387.92	4.28*

Monophyly constraints: A, best trees without any constraints; B, Entomobryidae + Paronellidae sensu Soto-Adames *et al.*; C, Entomobryidae + (Paronellidae + Cyphoderidae); D, (Orchesellinae + Heteromurinae) + remaining taxa; E, Heteromurinae + (Orchesellinae + remaining taxa); F, “Entomobryinae” + (Seirinae + “Lepidocyrtinae”). ** and * respectively represent very strong and strong evidence against an alternative hypothesis.

**Table 3:** Phylogenetic signal tests for each morphological character on a ML tree and a BI consensus tree.

Character	**ML Tree**				**BI Tree**			
	**ri**	**obs**	**permu**	**obs/permu**	**ri**	**obs**	**permu**	**obs/permu**
Dens	0.375	6	6	1.000	0.375	6	6	1.000
Mucro	0.625	6	9	0.667	0.625	6	9	0.667
Scales	0.579	9	12	0.750	0.579	9	13	0.690
Tergal S-chaetae	1.000	9	22	0.409	0.941	10	22	0.455
S-chaetae on thorax	0.933	5	13	0.385	0.867	6	13	0.462
S-chaetae on Abd. I	0.938	4	13	0.308	0.875	5	13	0.385
S-chaetae on Abd. II	0.929	5	15	0.333	0.929	5	15	0.333
S-chaetae on Abd. III	0.857	6	15	0.400	0.857	6	14	0.429
S-chaetae on Abd. V	1.000	4	8	0.444	1.000	4	9	0.444

The retention index (ri) and the method of Maddison and Slatkin^24^ are employed for the tests. obs, observed number of character transitions; permu, permutation number of character transitions; S-chaetae, tergal specialized chaetae.
