# Supplementary material for: Molecular phylogeny supports S-chaetae as a key character better than jumping organs and body scales in classification of Entomobryoidea (Collembola)
Source: Sci Rep. 2015 Jul 27;5:12471. doi: 10.1038/srep12471 (PMC4515636; doi:10.1038/srep12471)

# Molecular phylogeny supports S-chaetae as a key character better than jumping organ and body scales in classification of Entomobryoidea (Collembola)

Feng Zhang[1], Dan-Dan Sun[1], Dao-Yuan Yu[2] & Bei-Xin Wang[1]*

[1]Department of Entomology, College of Plant Protection, Nanjing Agricultural University, Nanjing 210095, P. R. China, [2]Department of Soil Sciences, College of Resources and Environmental Science, Nanjing Agricultural University, Nanjing 210095, P. R. China.

Correspondence and requests for materials should be addressed to B.-X.W.

(wangbeixin@njau.edu.cn and wangbeixin@gmail.com)

**Table S1.** Sequenced taxa, collection locality, and Genbank accession numbers. Numbers marked with an asterisk (*) are newly sequenced in the present study with others extracted from Zhang *et al.*[20].

| Group | Species name | Locality | 18S | 28S D1–3 | COI | 16S |
|---|---|---|---|---|---|---|
| **Tomoceridae** | *Tomocerus ocreatus* | China | KC236262 | KC236303 | KM978376* | KC236221 |
| **Isotomidae** | *Folsomia candida* | China | KC236239 | KC236281 | KM978353* | KC236200 |
| | *Folsomia qudrioculata* | France | KC236240 | KC236280 | KM978354* | KC236199 |
| **Orchesellinae** | *Ochesella cincta* | France | KC236250 | KC236290 | KM978365* | KC236208 |
| | *Orchesellides sinensis* | China | KC236251 | KC236293 | KM978363* | KC236209 |
| | *Orchesellides* sp. | China | KC236226 | KC236267 | KM978364* | KC236217 |
| | *Heteromurus major* | France | KC236241 | KC236282 | KM978355* | KC236201 |
| | *Heteromurus nitidus* | France | KC236242 | KC236283 | KM978356* | KC291493 |
| | *Dicranocentrus wangi* | China | KC236232 | KC236273 | KM978348* | KC236192 |
| | *Alloscopus* sp. | China | KM978398 | KM978335 | KM978344* | KM978389 |
| **Seirinae** | *Seira delamarei* | China | KC236255 | KC236292 | KM978370* | KC236213 |
| | *Seira barnardi* | South Africa | KC236254 | KC236296 | - | KC236212 |
| | *Seira* sp1 | China | KC236257 | KC236297 | KM978371* | KC236214 |
| | *Seira* sp2 | South Africa | KC236256 | KC236298 | KM978372* | KC236215 |
| **Lepidocyrtinae** | *Pseudosinella abla* | France | KC236253 | KC236295 | KM978368* | KC236211 |
| | *Pseudosinella tumula* | China | KC236252 | KC236294 | KM978367* | KC236210 |
| | *Lepidocyrtus* sp1 | China | KC236248 | KC236289 | KM978361* | KC236206 |
| | *Lepidocyrtus* sp2 | China | KC236249 | KC236291 | KM978362* | KC236207 |

| | | | | | | |
|---|---|---|---|---|---|---|
| | *Ascocyrtus* sp. | China | KC236228 | KC236269 | KM978345* | KC236190 |
| **Entomobryinae** | *Sinhomidia bicolor* | China | KC236260 | KC236301 | KM978375* | KC236220 |
| | *Willowsia japonica* | China | KC236265 | KC236307 | KM978378* | KC236224 |
| | *Willowsia guangdongensis* | China | KC236264 | KC236306 | KM978377* | KC236223 |
| | *Willowsia nigromaculata* | France | KC236263 | KC236304 | KM978379* | KC236222 |
| | *Willowsia* sp1 | China | KC236247 | KC236288 | KM978380* | KC236205 |
| | *Willowsia* sp2 | China | KC236266 | KC236305 | KM978396* | KC236225 |
| | *Willowsia* sp3 | China | KC236238 | KC236277 | KM978381* | KC236198 |
| | *Entomobrya proxima* | China | KC236236 | KC236279 | KM978351* | KC236197 |
| | *Entomobrya aino* | China | KC236235 | KC236279 | KM978350* | KC236195 |
| | *Entomobrya multifasciata* | France | KC236237 | KC236276 | KM978392* | KC236196 |
| | *Entomobrya* sp | China | KC236234 | KC236278 | KM978352* | KC236194 |
| | *Homidia sinensis* | China | KC236245 | KC236286 | KM978359* | KC236203 |
| | *Homidia socia* | China | KC236246 | KC236287 | KM978360* | KC236204 |
| | *Homidia sichuanensis* | China | KC236244 | KC236285 | KM978358* | - |
| | *Sinella curviseta* | China | KC236258 | KC236300 | KM978373* | KC236219 |
| | *Sinella longisensilla* | China | KC236259 | KC236299 | KM978374* | KC236216 |
| | *Coecobrya tenebricosa* | France | KC236231 | KC236272 | KM978347* | KC236191 |
| | *Drepanura* sp. | China | KC236233 | KC236274 | KM978349* | KC236193 |
| | *Himalanura* sp. | China | KC236243 | KC236284 | KM978357* | KC236202 |
| **Paronellidae** | *Akabosia matsudoensis* | China | KM978397* | KM978334 | KM978343* | KM978387* |
| Cremastocephalini | *Salina celebensis* | China | KM978404* | KM978341* | KM978394* | KM978388* |

| | | | | | | |
|---|---|---|---|---|---|---|
| | *Salina* sp. | China | KM978405* | KM978342* | KM978369* | KM978386* |
| Callyntrurini | *Callyntrura guangdongensis* | China | KM978399* | KM978336* | KM978391* | KM978382* |
| | *Callyntrura* sp. | China | KM978400* | KM978337* | KM978346* | KM978383* |
| | *Pseudoparonella tanimbarica* | New Caledonia | KM978403* | KM978340* | KM978393* | KM978384* |
| | *Pseudoparonella* sp. | New Caledonia | KM978402* | KM978339* | KM978366* | KM978385* |
| Paronellini | *Cyphoderopsis* sp. | China | KM978408* | - | KM978406* | KM978407* |
| **Cyphoderidae** | *Cyphoderus javanus* | China | KM978401* | KM978338* | - | KM978390* |

**Table S2.** Character states and coding and posterior probabilities of ancestral states summarized in MS Excel .xls format. ACSR were reconstructed using maximum parsimony (MP), maximum likelihood (ML) on 15,000 Bayesian posterior trees, as well as a fully Bayesian method (BayesTraits). MP and ML probabilities don't necessarily add to 1 because equivocal states were not counted. Bayesian analyses were performed under single-rate and unrestricted-rate models with Gamma-distributed priors seeded by shape parameter hyperpriors.

Figure S1. Ancestral state of thoracic S-chaetae in Entomobryoidea reconstructed over 15,000 posterior trees using ML method and shown on a Bayesian consensus tree. Each node indicates character states with different colorations and the proportion of the state over all examined trees.

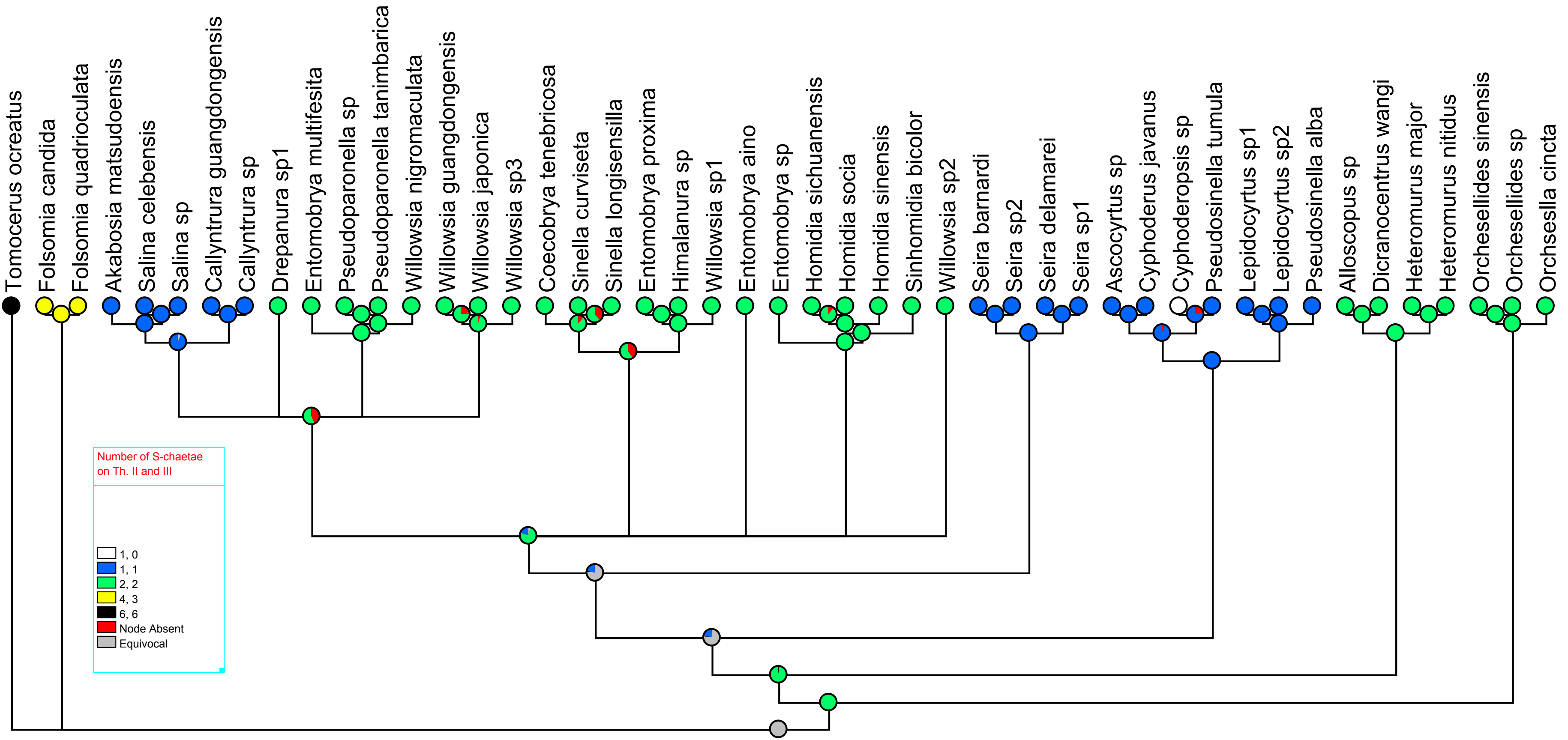

Figure S2. Ancestral state of S-chaetae on Abd. I in Entomobryoidea reconstructed over 15,000 posterior trees using ML method and shown on a Bayesian consensus tree. Each node indicates character states with different colorations and the proportion of the state over all examined trees.

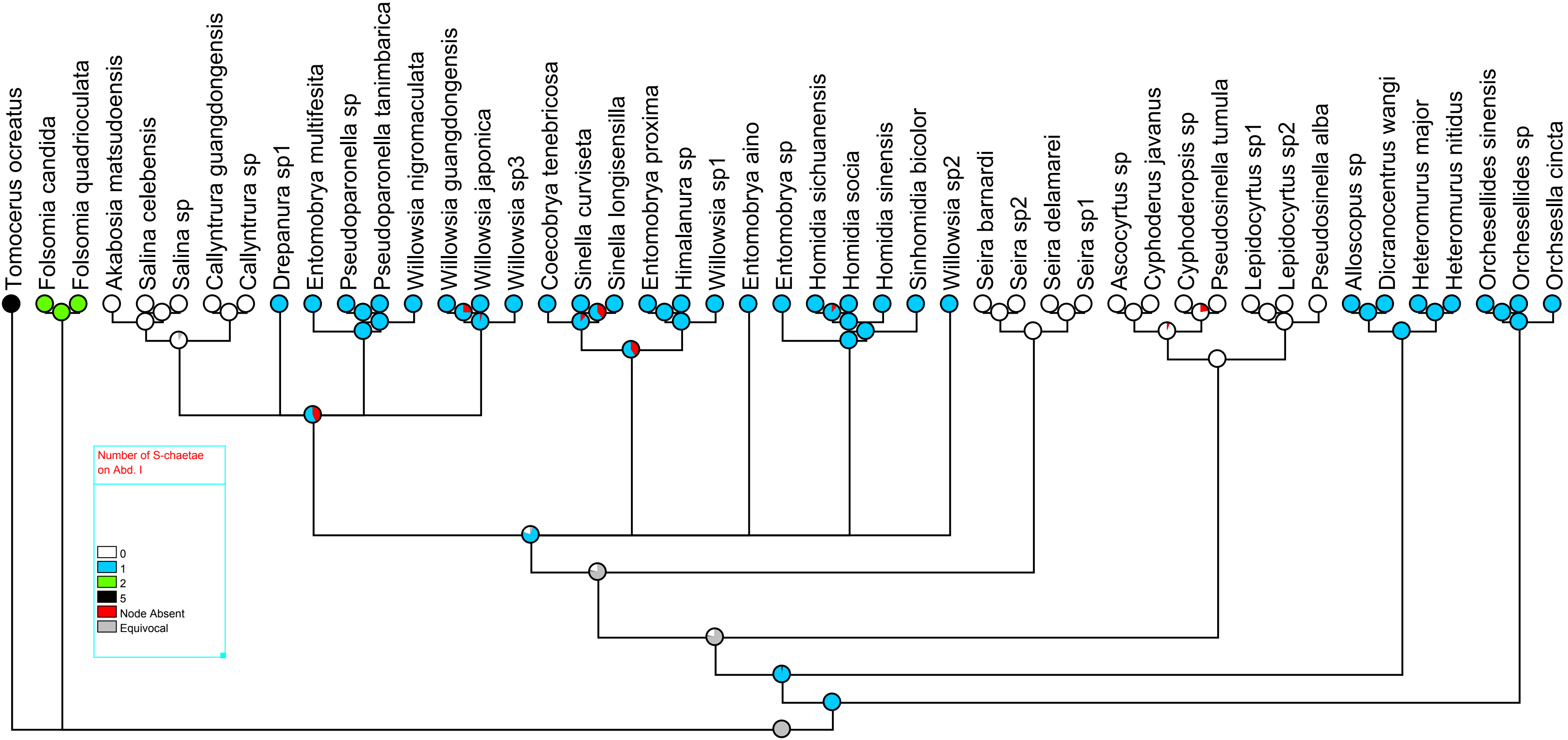

Figure S3. Ancestral state of S-chaetae on Abd. II in Entomobryoidea reconstructed over 15,000 posterior trees using ML method and shown on a Bayesian consensus tree. Each node indicates character states with different colorations and the proportion of the state over all examined trees.

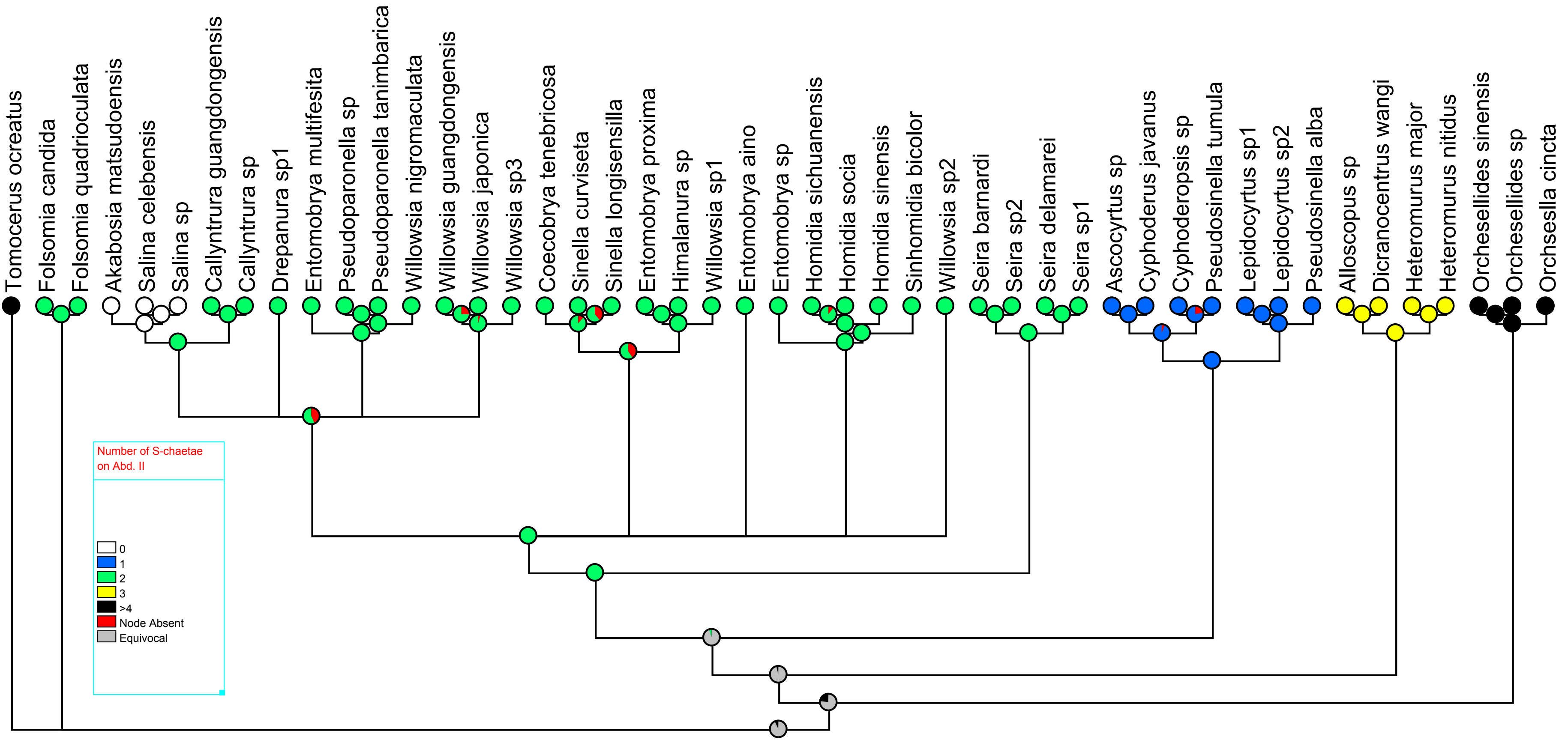

Figure S4. Ancestral state of S-chaetae on Abd. III in Entomobryoidea reconstructed over 15,000 posterior trees using ML method and shown on a Bayesian consensus tree. Each node indicates character states with different colorations and the proportion of the state over all examined trees.

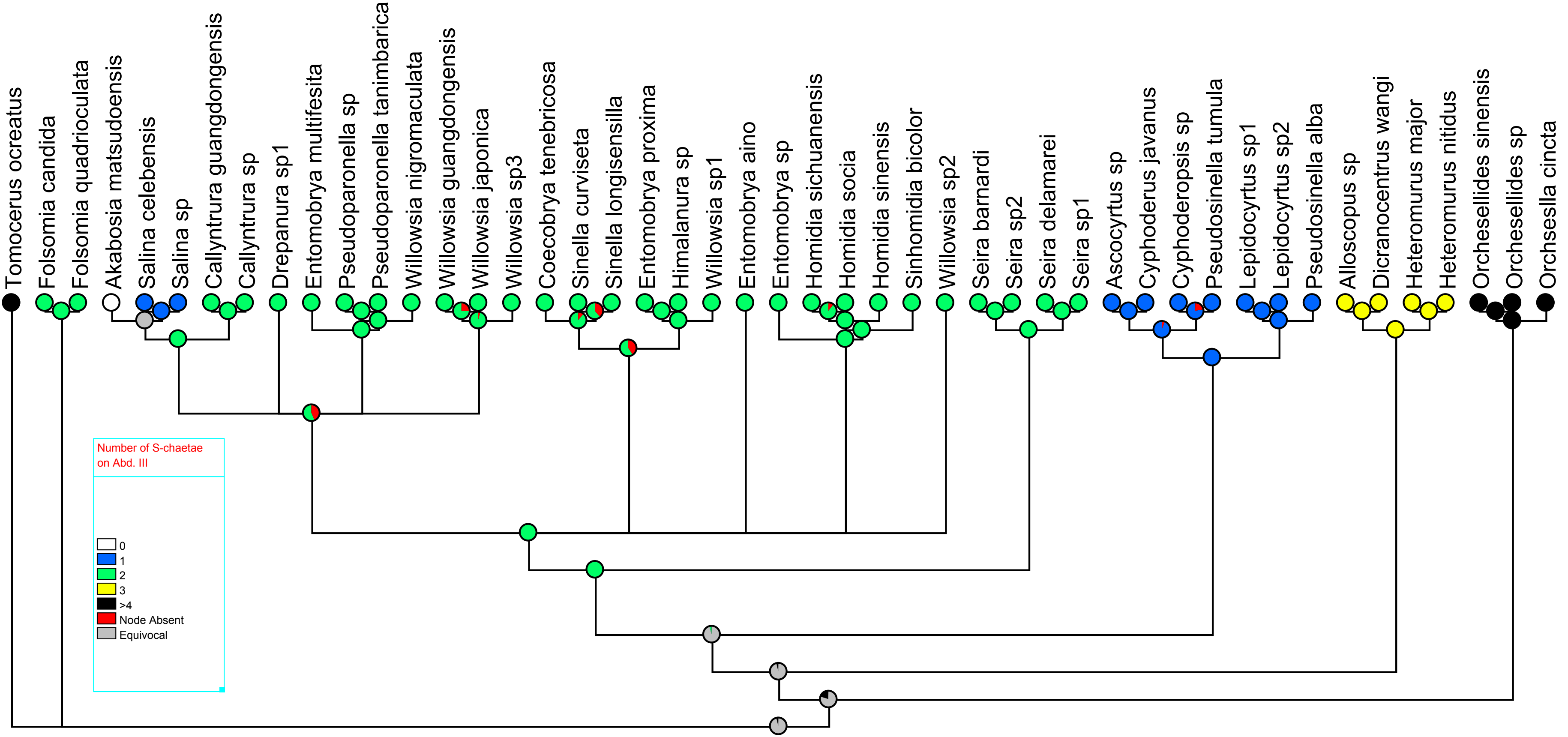

Figure S5. Ancestral state of S-chaetae on Abd. V in Entomobryoidea reconstructed over 15,000 posterior trees using ML method and shown on a Bayesian consensus tree. Each node indicates character states with different colorations and the proportion of the state over all examined trees.

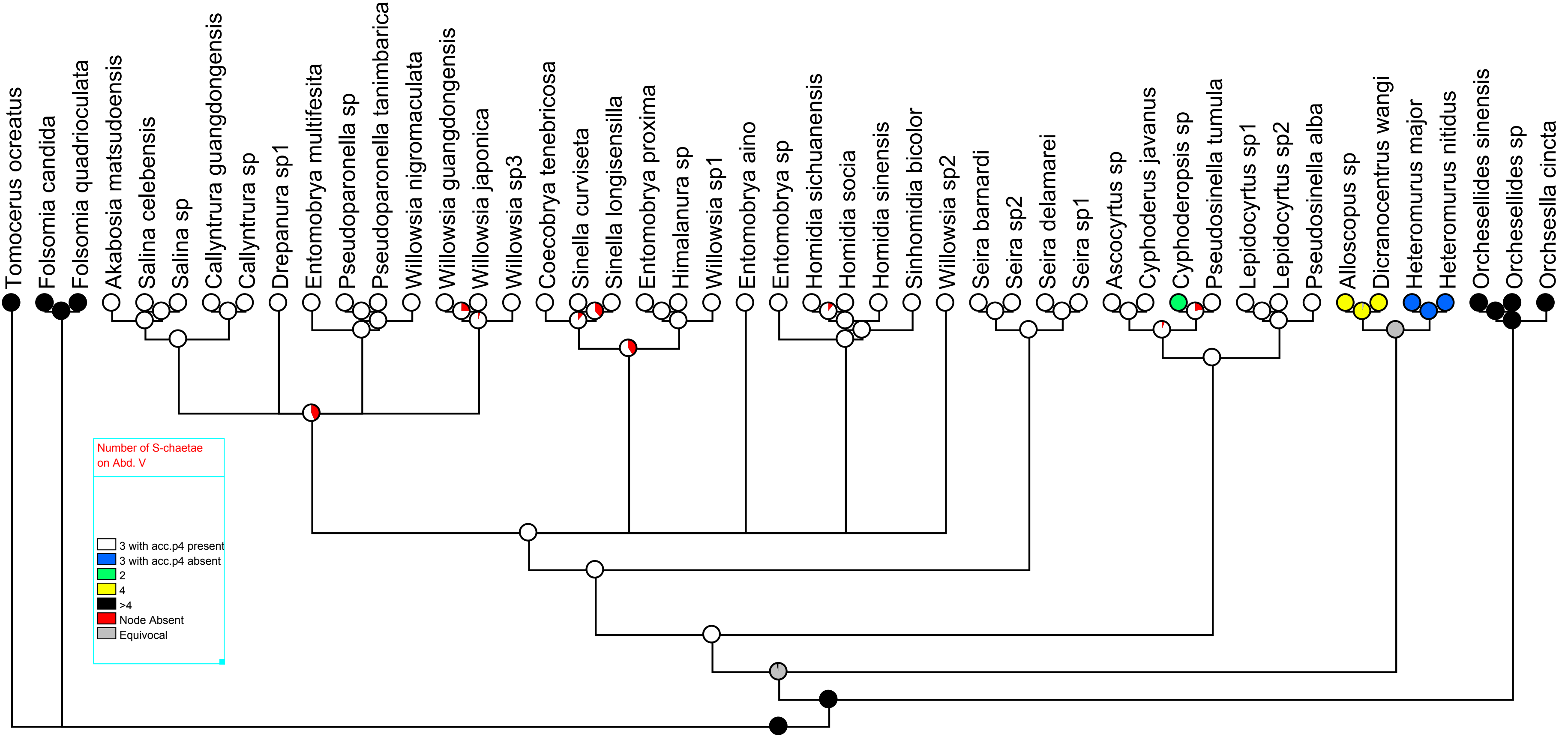

Supplement: Supplementary Information [file srep12471-s1.pdf]
